# Persistent Diarrhea and Eosinophilic Esophagitis Resulting From Chronic Cannabidiol Usage for Refractory Epilepsy

**DOI:** 10.1097/PG9.0000000000000253

**Published:** 2022-09-30

**Authors:** Maitri Patel, Jeremy P. Middleton, Howard P. Goodkin, Barrett Barnes, Emily McGowan, Ryan Eid

**Affiliations:** From the * School of Medicine, University of Virginia, Charlottesville, VA; † Department of Pediatrics, University of Virginia, Charlottesville, VA; ‡ Department of Neurology and Pediatrics, University of Virginia, Charlottesville, VA; § Division of Pediatric Gastroenterology, University of Virginia School of Medicine, Charlottesville, VA; ∥ Division of Allergy and Immunology, University of Virginia School of Medicine, Charlottesville, VA; ¶ Division of Allergy and Clinical Immunology, Johns Hopkins University School of Medicine, Baltimore, MD.

**Keywords:** chronic diarrhea, eosinophilic esophagitis, esophageal eosinophilia, cannabidiol, tetrahydrocannabinol, cannabinoid receptor 1, cannabinoid receptor 2, transient receptor potential cation channel subfamily V member 1, platelet activating factor

## Abstract

Cannabidiol is used in the care of treatment-resistant epilepsy. It has been associated with varying side effects, ranging from somnolence to diarrhea and weight loss. We present a patient on chronic cannabidiol therapy who had persistent diarrhea, abdominal pain, weight loss, and esophageal eosinophilia that improved with cannabidiol dose adjustment.

## INTRODUCTION

In 2018, the US Food and Drug Administration (FDA) approved the oil-based cannabidiol extract Epidiolex (cannabidiol; Greenwich Biosciences, Jazz Pharmaceuticals, Dublin, Ireland) for treatment of seizures associated with Lennox-Gastaut and Dravet syndrome and in 2020 expanded its approval for patients with tuberous sclerosis complex (1). Open-label usage of cannabidiol has also been utilized in patients with CDKL5 disorder and Aicardi, Doose (Myoclonic Astatic Epilepsy), and Dup15q syndromes (2). Cannabidiol (CBD), unlike Δ9-tetrahydrocannabinol (THC), is a nonpsychoactive cannabinoid that has anticonvulsant properties with an incompletely understood mechanism. Since its approval, cannabidiol has been successfully used for treating refractory epilepsy, although it does have a wide range of adverse side effects ranging from somnolence and anorexia to diarrhea and weight loss (1). We report a patient with Aicardi syndrome on chronic cannabidiol therapy who presented with persistent diarrhea, abdominal pain, peripheral eosinophilia, esophageal eosinophilia, and weight loss that resolved after cannabidiol dose reduction.

## CASE REPORT

A 13-year-old female with Aicardi syndrome and treatment-resistant epilepsy presented to the emergency department with several weeks of nonbloody diarrhea, abdominal distention, and vomiting. Review of systems was otherwise unremarkable, and she had no sick contacts, recent medication, or diet changes, travel history, or pathogen exposure. Physical exam was notable for hyperactive bowel sounds and intermittent discomfort with palpation in the right upper quadrant and epigastrium. On past medical history, she had global developmental delay, feeding difficulties requiring gastrostomy tube feeds, and treatment-refractory epilepsy for which she had a vagal nerve stimulator and was treated with cannabidiol, clobazam, felbamate, and levetiracetam. Cannabidiol therapy was initiated in 2016 using a product called Haleigh’s Hope (CBD oil; Haleigh’s Hope, Commerce City, CO) but was transitioned to the FDA-approved form of cannabidiol in 2018 with a recent dose escalation 3 months prior.

Initial evaluation in the emergency department revealed an unremarkable, Comprehensive Metabolic Panel (CMP), COVID-19 Polymerase Chain Reaction (PCR), *Clostridium difficile* PCR, ova and parasite test, multiplex PCR assay for gastrointestinal pathogens, and celiac panel. Absolute eosinophil count was 650 k/µL, C-reactive protein was 2.1 mg/dL, and stool was hemoccult positive with calprotectin of 2530 µg/g. She was hospitalized for intravenous hydration and consultation with pediatric gastroenterology. Despite a negative infectious evaluation, she was discharged on a 10-day course of metronidazole as preemptive coverage for inflammatory diarrhea until further outpatient workup.

At follow-up 10 days after hospitalization, she continued to have persistent diarrhea with intermittent hematochezia, feeding intolerance, abdominal pain, and weight loss. She underwent cross-sectional computer tomography (CT) imaging and upper endoscopy and colonoscopy with biopsy. The abdominal CT was normal with no bowel wall thickening. Upper endoscopy was unremarkable, but colonoscopy revealed small superficial ulcers and erythematous plaques in her rectum and sigmoid colon. Biopsies revealed >40 eosinophils per high-powered field in her distal, mid, and proximal esophagus; colonic biopsies were normal with no evidence of inflammation or viral effect, suggesting that her hematochezia was likely secondary to perianal irritation.

Although eosinophilic esophagitis (EoE) was not thought to be the cause of her persistent diarrhea, because of the abnormal histopathology, she was treated with a proton pump inhibitor, which was eventually advanced to a 5-food elimination diet. However, she continued to have profuse diarrhea.

After 6 months of ongoing gastrointestinal symptoms with no distinct etiology, pediatric neurology recommended a gradual decrease of the cannabidiol from 20 mg/kg/day (450 mg BID) to 15 mg/kg/day (350 mg BID). Over the next 2 weeks, the diarrhea and abdominal pain resolved, and the patient quickly regained the 8 kg she had lost. Fecal calprotectin declined to 197 µg/g, and repeat upper endoscopy, colonoscopy and video capsule endoscopy demonstrated complete resolution of her esophageal eosinophilia. Her peripheral eosinophilia decreased to 110 k/µL. She remains on cannabidiol 10 mg/kg/day and has not had any further gastrointestinal issues.

## DISCUSSION

Our case of a child with Aicardi syndrome and treatment-resistant epilepsy on longstanding cannabidiol had resolution of chronic diarrhea with dose reduction of cannabidiol. Although the use of cannabidiol decreases seizure frequency, up to 79% of patients may experience mild adverse effects, including diarrhea, somnolence, decreased appetite, and increased aminotransferases (1,3,4). A 2-year study observing the long-term safety and efficacy of cannabidiol in both adults and children reported 737 episodes of diarrhea out of 3042 adverse events among 169 total participants (3). Additionally, in the largest open-label interventional trial of cannabidiol for treatment-resistant epilepsy, subjects receiving 15 mg/kg/day or higher were 4.5 times more likely to have gastrointestinal side effects including diarrhea, abdominal pain and anorexia (5).

The proposed mechanism of action of cannabidiol causing gastrointestinal side effects is still unclear. Historically, cannabinoids have typically been associated with anti-diarrheal properties. Cannabinoids play a role in the endocannabinoid system (ECS), a physiologic system, which regulates homeostasis of various organ systems, including the gastrointestinal tract. Within the ECS, G protein-coupled cannabinoid receptors 1 and 2 (CB1 and CB2) interact with endogenous endocannabinoids and with externally consumed cannabinoids to play a key role in gut motility homeostasis and minimization of gastrointestinal inflammation (6). In addition, CBD signaling through transient receptor potential cation channel subfamily V member 1 (TRPV1) in the esophagus has bene shown to promote eosinophilic inflammation (Supplemental Digital Content Figure 1, http://links.lww.com/PG9/A91) (7).

**Figure:**
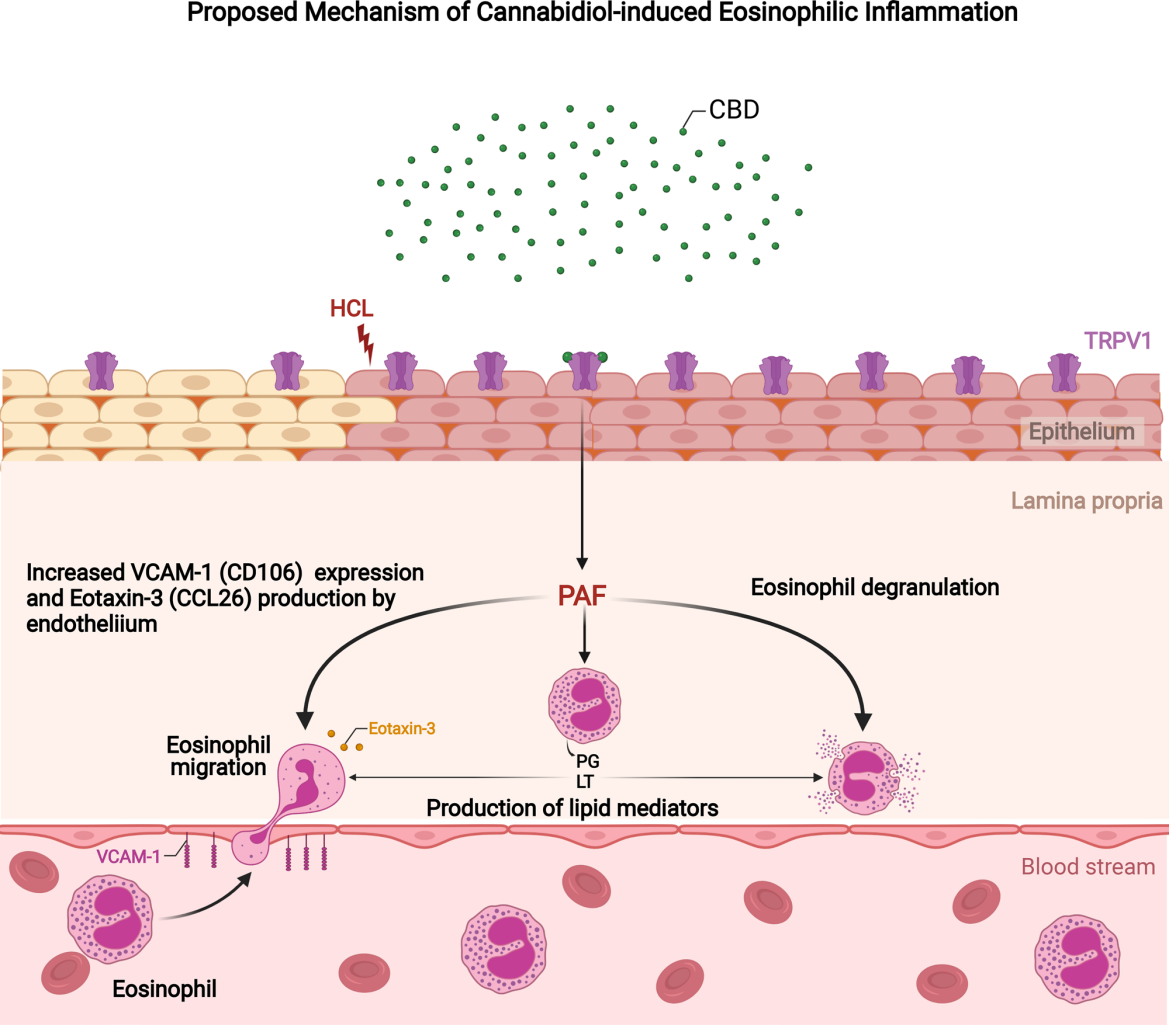


Other proposed mechanisms of cannabinoid-induced gastrointestinal side effects are possible reaction to the inactive components in this product—sesame seed oil, strawberry flavor, and sucralose. However, an allergic reaction to the sesame oil or osmotic effect from the sucralose causing the diarrhea are unlikely due to an increase in side effects with dosage increase and the relatively small volumes of medication delivered.

Although higher doses appear to be strongly associated with increased gastrointestinal adverse events, our patient tolerated a dose increase to 20 mg/kg/day of cannabidiol for 3 months prior to the onset of her gastrointestinal issues. It is possible our patient had an infectious insult that potentiated the gastrointestinal effects of the cannabidiol perhaps by disrupted barrier function or altered mucosal metabolism. As the gut microbiome has direct and indirect effects on drug metabolism, it is also possible that a post infectious dysbiosis may have affected how our patient reacted to the prescribed cannabidiol (8).

This case highlights some of the significant side effects that can be seen in patients using cannabidiol and describes the first case of CBD exacerbated esophageal eosinophilia. In other disorders such as irritable bowel syndrome, depression or Parkinson’s Disease, researchers continue to seek additional therapeutic applications for cannabidiol (9). As physicians move forward embracing the novelties of this new class of drugs, it is important to keep these adverse side effects in mind.

## ACKNOWLEDGMENTS

The patient’s parents provided informed consent for publication of the details of this case.

## Supplementary Material


