# Supplementary material for: Persistent Diarrhea and Eosinophilic Esophagitis Resulting From Chronic Cannabidiol Usage for Refractory Epilepsy
Source: JPGN Rep. 2022 Sep 30;3(4):e253. doi: 10.1097/PG9.0000000000000253 (PMC10158314; doi:10.1097/PG9.0000000000000253)
Supplement: Supplementary file 1 [file pg9-3-e253-s001.pdf]

Figure 1: Proposed mechanism underlying cannabidiol-induced esophageal eosinophilia. The expression of the transient receptor potential cation channel subfamily V member 1 (TRPV1) is increased on the surface of epithelial cells in the presence of chronic exposure to hydrochloric acid (HCL). Cannabidiol (CBD) signaling through TRPV1 leads to the production of platelet activating factor (PAF) and several downstream pro-eosinophilic signals. Leukotrienes (LT). Prostaglandins (Pg). This image was created with BioRender.com
